# Expression of the Carbohydrate Lewis Antigen, Sialyl Lewis A, Sialyl Lewis X, Lewis X, and Lewis Y in the Placental Villi of Patients With Unexplained Miscarriages

**DOI:** 10.3389/fimmu.2021.679424

**Published:** 2021-05-31

**Authors:** Zhi Ma, Huixia Yang, Lin Peng, Christina Kuhn, Anca Chelariu-Raicu, Sven Mahner, Udo Jeschke, Viktoria von Schönfeldt

**Affiliations:** ^1^ Department of Obstetrics and Gynaecology, University Hospital LMU Munich, Munich, Germany; ^2^ Department of Obstetrics and Gynaecology, University Hospital Augsburg, Augsburg, Germany

**Keywords:** Lewis antigen, ST3GAL6, NEU1, villous vessel, unexplained miscarriage

## Abstract

**Background:**

Lewis antigens such as Sialyl Lewis A (sLeA), Sialyl Lewis X (sLeX), Lewis X (LeX), and Lewis Y (LeY) are a class of carbohydrate molecules that are known to mediate adhesion between tumor cells and endothelium by interacting with its selectin ligands. However, their potential role in miscarriage remains enigmatic. This study aims to analyze the expression pattern of sLeA, sLeX, LeX, and LeY in the placental villi tissue of patients with a medical history of unexplained miscarriages.

**Methods:**

Paraffin-embedded slides originating from placental tissue were collected from patients experiencing a miscarriage early in their pregnancy (6–13 weeks). Tissues collected from spontaneous (n = 20) and recurrent (n = 15) miscarriages were analyzed using immunohistochemical and immunofluorescent staining. Specimens obtained from legally terminated normal pregnancies were considered as control group (n = 18). Assessment of villous vessel density was performed in another cohort (n = 10 each group) of gestation ages-paired placenta tissue. Protein expression was evaluated with Immunoreactive Score (IRS). Statistical analysis was performed by using Graphpad Prism 8.

**Results:**

Expression of sLeA, sLeX, LeX, and LeY in the syncytiotrophoblast was significantly upregulated in the control group compared with spontaneous and recurrent miscarriage groups. However, no prominent differences between spontaneous and recurrent miscarriage groups were identified. Potential key modulators ST3GAL6 and NEU1 were found to be significantly downregulated in the recurrent miscarriage group and upregulated in the spontaneous group, respectively. Interestingly, LeX and LeY expression was also detected in the endothelial cells of villous vessels in the control group but no significant expression in miscarriage groups. Furthermore, assessment of villous vessel density using CD31 found significantly diminished vessels in all size groups of villi (small villi <200 µm, *P* = 0.0371; middle villi between 200 and 400 µm, *P* = 0.0010 and large villi >400 µm, *P* = 0.0003). Immunofluorescent double staining also indicated the co-localization of LeX/Y and CD31.

**Conclusions:**

The expression of four mentioned carbohydrate Lewis antigens and their potential modulators, ST3GAL6 and NEU1, in the placenta of patients with miscarriages was significantly different from the normal pregnancy. For the first time, their expression pattern in the placenta was illustrated, which might shed light on a novel understanding of Lewis antigens’ role in the pathogenesis of miscarriages.

## Introduction

Miscarriage is the most common complication of pregnancy, which affects around 9–20% of clinically confirmed pregnancies. In addition, if biochemical loss due to implantation failure is considered, this rate can reach up to 50% (1). The establishment of a healthy pregnancy implies interaction between the embryonal structure and endometrium. Any alterations within this process may trigger miscarriages: chromosomal errors, anatomical uterine defects, autoimmune dysregulations, and endometrial abnormality. Prior studies have shown that more than 50% of cases of miscarriages are associated with the above-mentioned alterations, however the remaining causes are unexplained (2). According to the guidelines of the European Society of Human Reproduction and Embryology (ESHRE) and the American Society for Reproductive Medicine (ASRM), recurrent miscarriage (RM) is defined as the failure of two or more clinically confirmed pregnancies, excluding ectopic and molar pregnancies (3, 4). The average prevalence of recurrent miscarriage is estimated to be between 1 and 4% (1). Most miscarriages, including spontaneous or recurrent events, occur in the first trimester of pregnancy (5).

**Figure 1:**
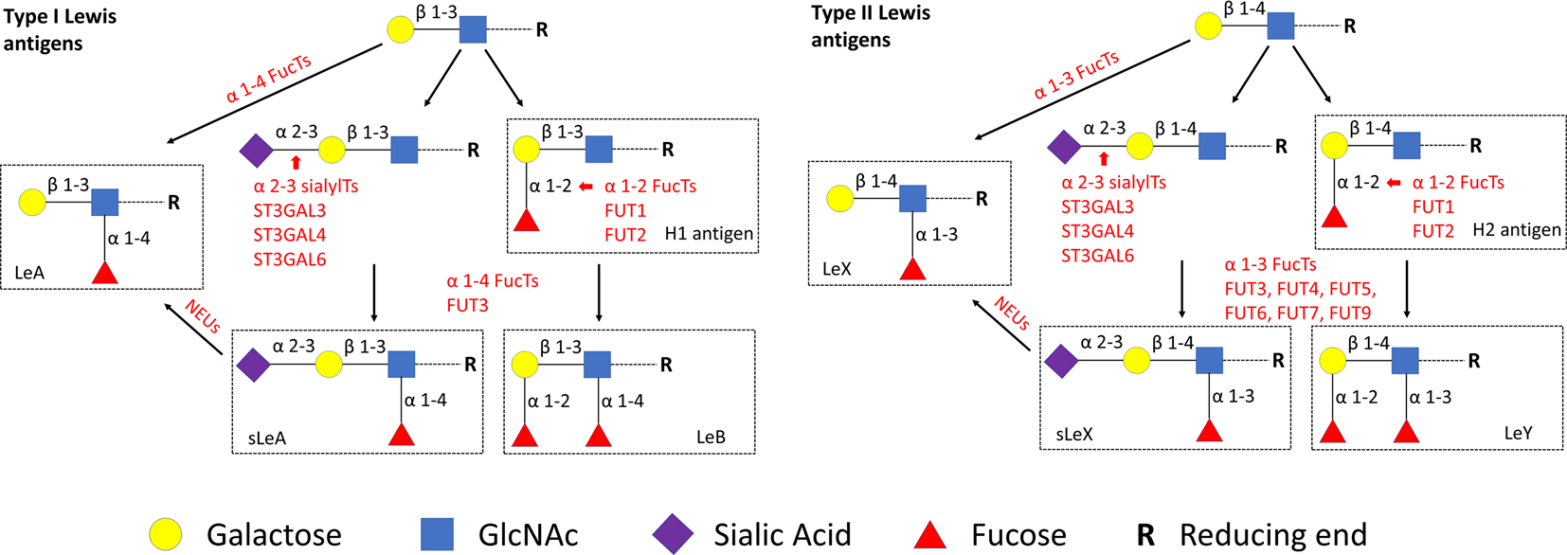
Lewis antigens biosynthetic pathways. GlcNAc, N-acetylglucosamine. NEUs, Neuraminidases.

Lewis antigens are series of carbohydrate epitopes with terminal fucosylation (6). According to different glycosidic bonds, Lewis antigens are classified as type I [H1 antigen, Lewis A (LeA), Lewis B (LeB) and Sialyl Lewis A (sLeA)] and type II [H2 antigen, Lewis X (LeX), Lewis Y (LeY) and Sialyl Lewis X (sLeX)], as shown in **Figure 1**. Lewis antigens are known to mediate adhesion between tumor cells and endothelium by interacting with their selectin ligands. Furthermore, upregulated expression of Lewis antigens has been reported in many types of cancers (7). More recently, it was showed that Lewis epitopes are also involved in early embryogenesis and later development of the pregnancy. Specifically, sLeX participates in the process of sperm–zona pellucida binding (8). During the menstrual cycle, the expression of sLeX in the human endometrium is temporally regulated, reaching the highest level during embryo implantation (9). LeY was also shown to be involved in a cellular model of trophoblast attachment to the epithelium (10). Given the involvement of Lewis antigens and their selectin ligands in the initial steps in implantation (11), we speculate that Lewis antigens may also have potential significance in the pathogenesis of miscarriages.

## Methods

### Patient

Placenta samples of recurrent miscarriages (RM, n = 15), spontaneous miscarriages (SM, n = 20), and normal pregnancies (NC, n = 18) were chosen from the tissue bank of the Department of Obstetrics and Gynecology of LMU Munich. The details including both patients’ inclusion and exclusion criteria and the process of samples acquisition have been described in our previous work (12). **Supplementary Figure 1** reflects the gestational ages distribution in different groups of samples. **Table 1** summarizes the demographic and clinical characteristics of the study population.

**Table 1:** Demographic and clinical characteristics of the study population.

Characteristics	Normal Control n = 18	Recurrent Miscarriage n = 15	Spontaneous Miscarriage n = 20	*P* value
Maternal age (years)	31.28 ± 6.03 (21–43)	34.60 ± 5.96 (22–42)	34.35 ± 4.72 (26–42)	0.14
Gestational age (weeks)	9.04 ± 1.90 (6–13)	9.65 ± 1.66 (7–12)	9.45 ± 1.54 (6–12)	0.57
Gravidity	3.28 ± 1.67 (1–6)	3.27 ± 1.34 (2–6)	1.60 ± 0.82 (1–4)	0.0002
Parity	1.15 ± 1.10 (0–4)	0.80 ± 0.94 (0–3)	0.45 ± 0.76 (0–3)	0.0035

Values are presented as Mean ± S.D.; the range is shown in parentheses.

### Gene Expression and Network Analysis

Enrichment of glycosylation and inflammatory response related signatures in RM patients were demonstrated by Gene set enrichment analysis (GSEA) (13) using two gene sets (GO_GLYCOSYLATION, GO:0070085 and HALLMARK_INFLAMMATORY_RESPONSE, M5932) and GSE76862 (14), The functional enrichment was carried out by using the GSEA method based on the Kyoto Encyclopedia of Genes and Genomes (KEGG) analyses with the clusterProfiler package of R (*P <*0.05 and FDR <0.25). To define the possible crosstalk of glycosylation with immune and adhesion process, differentially regulated GO_GLYCOSYLATION genes in RM were extracted and processed with a web-based tool of Metascape (https://metascape.org) (15) to get significantly enriched Gene Ontology (GO) biological processes and pathways network visualization.

### Immunohistochemistry

Paraffin-embedded slides of placenta tissue were dewaxed in xylol for 20 min and rinsed with 100% ethanol for the staining process. For inhibition of the endogen peroxidase reaction, slides were incubated in methanol with 3% H_2_O_2_ for 20 min followed by rehydration in descending ethanol gradients. Slides were then heated in a pressure pot containing a sodium citrate buffer (pH = 6.0), which consisted of 0.1 mM sodium citrate and 0.1 mM citric acid in distilled water. After cooling in distilled water and rinsing twice in PBS, all slides were incubated with a blocking solution (Reagent 1, ZytoChem Plus HRP Polymer System (mouse/rabbit), Zytomed Berlin, Germany) for 5 min to avoid non-specific binding.

Primary antibody incubation of every slide was performed for 16 h at 4°C, details are shown in **Table 2**. PBS (pH = 7.4) washing was applied between each step. Subsequently, 20 min incubation with post block (Reagent 2) and 30 min with HRP polymer (Reagent 3) were done according to the manufacturer’s protocol. Immunostaining was visualized with the DAB chromogen-substrate staining system (Dako, Denmark), the reaction was stopped with distilled water. Slides were counterstained with Hemalaun for 2 min, blued in tap water for 5 min, and rehydrated in an ascending ethanol gradient. Finally, all tissue slides were covered with Eukitt^®^ quick hardening mounting medium (Sigma Aldrich, USA). Negative controls were performed by replacing the primary antibodies with certain species-specific isotype control antibodies (Dako).

**Table 2:** Antibodies used in this study.

Antibody	Isotype	Clone	Dilution	DAB time	Source
sLeA	Mouse IgG	Monoclonal	1:80 in PBS	30 s	Calbiochem
sLeX	Mouse IgM	Monoclonal	1:200 in PBS	5 min	BD Pharmingen
LeX	Mouse IgM	Monoclonal	1:200 in PBS	5 min	Novocastra
LeY	Mouse IgM	Monoclonal	1:50 in PBS	6 min	LSBio
CD31	Rabbit IgG	Polyclonal	1:50 in PBS	1 min	Abcam
Cy2	Goat IgG anti Mouse	Polyclonal	1:100 in Dako Antibody Diluent	–	Dianova
Cy3	Goat IgG anti Rabbit	Polyclonal	1:500 in Dako Antibody Diluent	–	Dianova
FUT4	Rabbit IgG	Polyclonal	1:100 in PBS	30 s	Prosci
ST3GAL3	Rabbit IgG	Polyclonal	1:500 in PBS	2 min	Invitrogen
ST3GAL4	Rabbit IgG	Polyclonal	1:100 in PBS	2 min	Sino Biological
ST3GAL6	Rabbit IgG	Polyclonal	1:100 in PBS	30 s	Novus Biologicals
NEU1	Rabbit*	Polyclonal	1:50 in PBS	3 min	Sigma-Aldrich

*Isotype is not mentioned in the datasheet.

The staining results were analyzed under the microscope Leitz (Wetzlar, Germany; Type 307-148.001 514686) by two independent observers. Intensity and distribution patterns of antigens’ expression were evaluated with immunoreactive score (IRS), the semi-quantitative score is calculated as the optical staining intensity (grades: 0 = none, 1 = weak, 2 = moderate, 3 = strong staining) multiplied by the total percentage of positively stained cells (0 = none, 1 ≤10%, 2 = 11–50%, 3 = 51–80% and 4 ≥ 81% of the cells). This multiplication has a minimum of 0 and a maximum of 12.

### Immunofluorescence

To identify the expression pattern of LeX and LeY in the endothelial cells of villous vessels, double immunofluorescence staining was performed in the specimens of healthy controls. All used antibodies are listed in **Table 2**. CD31 was used as a specific marker for endothelial cells. The same experimental steps were carried out as for immunohistochemistry until the step of blocking: slides were blocked with Ultra V Block solution (Lab Vision, USA) for 15 min and then incubated with specific primary antibodies for 16 h at 4°C. After washing twice in PBS, slides were incubated with Cy2-/Cy3-labeled fluorescent secondary antibodies (Dianova, Germany) for 30 min at room temperature in darkness to avoid fluorescence quenching. Finally, slides were embedded in Vectashield^®^ mounting medium with DAPI (Vector Laboratories, USA) for blue staining of the nucleus after washing and drying. A Fluorescent Axioskop photomicroscope (Zeiss, Germany) was used to examine all slides. Pictures were taken using a digital Axiocam camera system (Zeiss, Germany).

### Evaluation of Villous Vessels in Miscarriage and Control Groups

Another cohort (n = 10 each group) of gestation ages-paired placenta tissues was selected to perform CD31 staining, which visualized the villous vessels. Slides were evaluated by two independent observers using the microscope (Leica, Germany) at a magnification of ×100. Five fields were randomly captured for every slide. Any brown-staining endothelial cell or endothelial cell cluster that was clearly separate from adjacent vessels was considered as a single, countable vessel. The visualization of the vessel lumens was not a prerequisite for a structure to be identified as a vessel (16). To balance the significantly different vessel numbers between large and small villi, all villi were divided into three groups according to their diameter: small villi (<200 µm), middle villi (200–400 µm), and large villi (>400 µm). The final “number of vessels per villous” was calculated (number of vessels/number of villi of every sample) and statistically analyzed in different size groups, respectively.

### Statistical Analysis

Statistical differences between experimental groups were analyzed using Graphpad Prism 8 (Graphpad Software Inc., USA). Data in this study were represented as the mean ± SD for quantitative variables. The Gaussian distribution of the continuous variables was tested by the Kolmogorov–Smirnov statistic. Two-tailed Student’s paired *t*-test or Wilcoxon matched-pairs signed rank test was performed for the paired cohort. One way ANOVA or Kruskal–Wallis test was used for more than two comparison groups, and Dunnett’s test for multiple comparisons. Correlation analysis was performed using Spearman’s rank correlation coefficient. A *P* value <0.05 was considered to be statistically significant.

## Results

### Aberrant Glycosylation and Inflammatory Response in RM

**Figure 2:**
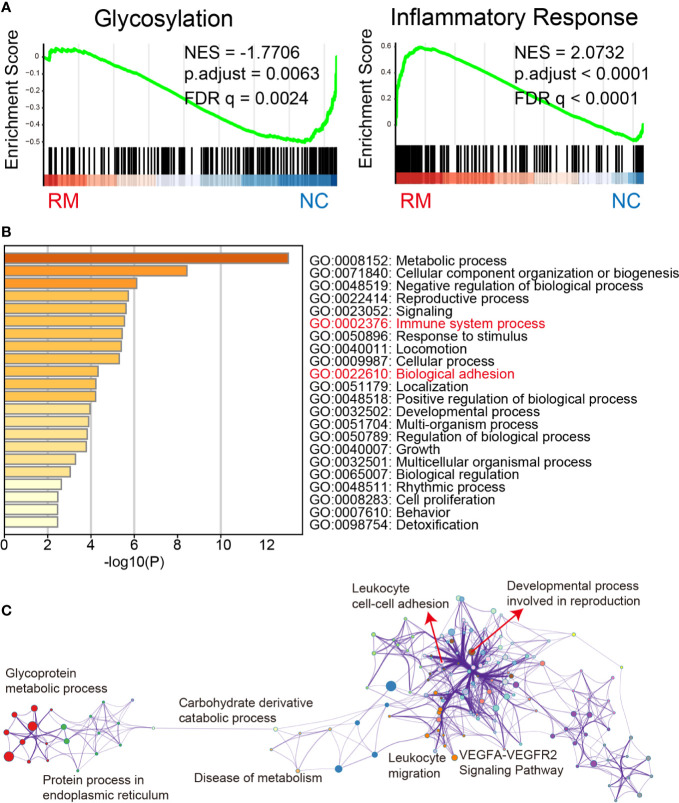
Gene expression and network analysis. **(A)** Gene Set Enrichment Analysis of glycosylation and inflammatory response related genes in the RM patients from previously published data set GSE76862. Shown are the graphic enrichment of two gene sets (GO_GLYCOSYLATION, GO:0070085 and HALLMARK_INFLAMMATORY_RESPONSE, M5932) in RM patients (left, red) compared with healthy controls (right, blue). NES, Normalized enrichment score; FDR, False discovery rate. **(B)** Metascape results obtained with the differentially downregulated GO_GLYCOSYLATION genes confirm that Immune system process and Biological adhesion are significantly related to the aberrant glycosylation status in RM. **(C)** Network visualization of pathways was enriched also with differentially downregulated GO_GLYCOSYLATION genes in RM by performing Metascape, different colors represent different GO term groups.

Our GSEA analysis indicated that glycosylation and inflammatory response were significantly changed in RM (**Figure 2A**). More specifically, glycosylation was downregulated (NES = −1.7706, FDR = 0.0024) and inflammatory response was upregulated (NES = 2.0732, FDR <0.0001). We next performed Metascape analysis including the significantly downregulated GO_GLYCOSYLATION genes in RM. Here, we identified distinctive GO biological processes “Immune system process and Biological adhesion”. These results might indicate their significant interaction with the downregulation of glycosylation status in RM (**Figure 2B**). Besides, KEGG pathway and GO enrichment analysis (**Supplementary Figure 2**) also revealed distinctive pathways and cellular components “Focal adhesion and Cell adhesion molecules”, with which Lewis antigens have been tightly associated (7). Furthermore, pathways network visualization (**Figure 2C**) indicated that leukocyte adhesion, migration, and developmental process might occur due to changes in glycoprotein metabolic process in RM. Notably, VEGFA-VEGFR2 signaling pathway, which plays the pivotal role in the angiogenesis in ovarian function and embryonic development (17), was also involved in the network.

### Downregulated Lewis Antigens in the Miscarriage Groups

**Figure 3:**
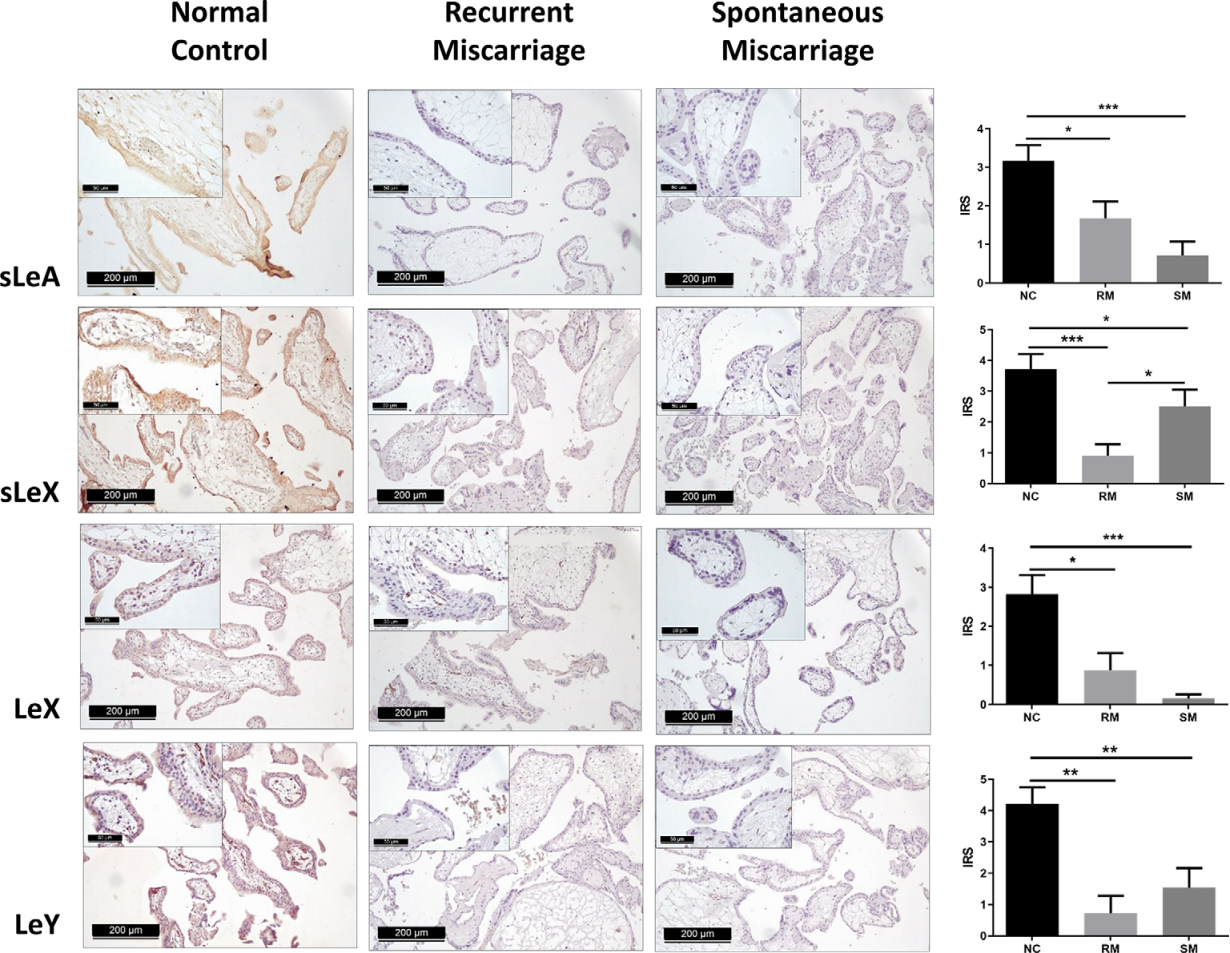
Immunohistochemical staining of four carbohydrate Lewis antigens in the syncytiotrophoblast of three groups of placenta tissues. *P < 0.05, **P < 0.01, ***P < 0.001.

**Figure 4:**
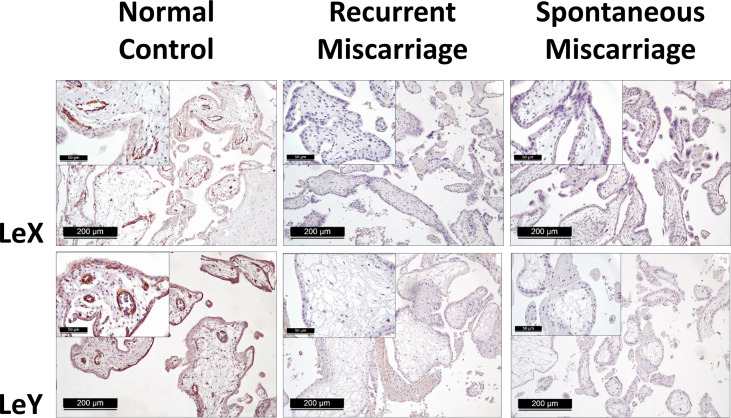
Immunohistochemical staining of LeX and LeY in the endothelial cells of villous vessels in three groups.

Positive expression of sLeA, sLeX, LeX, and LeY was detected in the syncytiotrophoblast across all groups included in this study (**Figure 3**). More specifically, sLeA was prominently lower in both RM and SM group than NC group (*P <*0.05 and *P <*0.001, respectively); sLeX expression was also prominently lower in both RM and SM group compared with NC group (*P <*0.001 and *P <*0.05, respectively) and sLeX was even lower in RM than SM group (*P <*0.05); LeX expression was significantly downregulated in both RM (*P <*0.05) and SM (*P <*0.001) groups comparing with NC group; LeY showed similar expression pattern to LeX in both RM (*P <*0.01) and SM (*P <*0.01) groups. Strikingly, LeX and LeY staining were also clearly identified inside the villi of the normal control group, probably the endothelial cells of villous vessels, but not significant in miscarriage groups (**Figure 4**).

**Figure 5:**
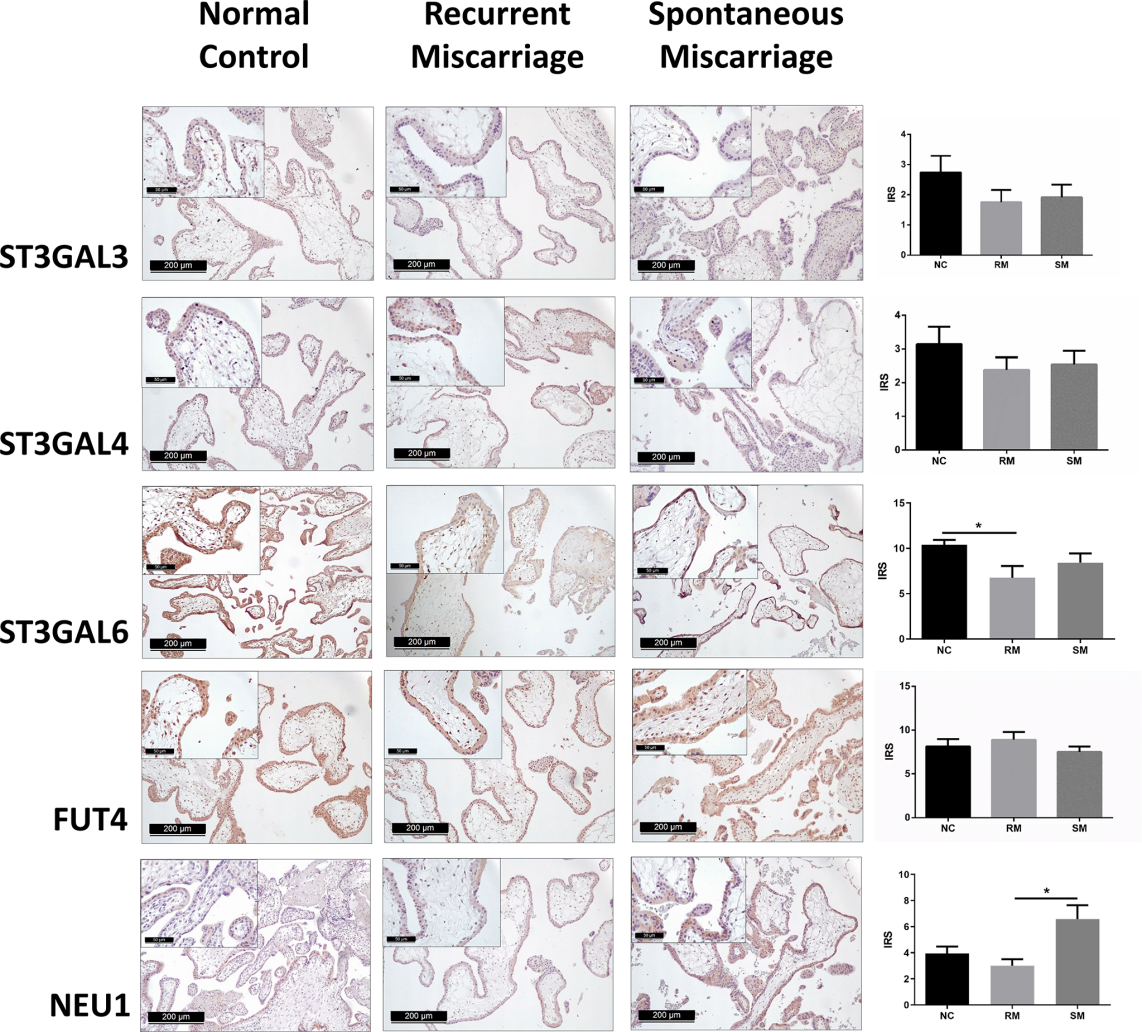
Immunohistochemical staining of five potential key enzymes that responsible for the synthesis of carbohydrate Lewis antigens in the syncytiotrophoblast of three groups of placenta tissues. *P < 0.05.

To identify potential key enzymes related to Lewis antigens in miscarriage, we evaluated the expression patterns of three α-2,3 sialyltransferases, ST3GAL3, -4, and -6, which act on the N-Acetyllactosamine structure (Galβ1,3/4GlcNAc) to create sLeA/X and related sialofucosylated glycans. Synthesis of Lewis antigens also requires the synergic action of variable fucosyltransferases (**Figure 1**), here α-1,3 fucosyltransferase FUT4 was investigated. Pretest staining of FUT3 and FUT6 (n = 5 each group) showed their strong expression in the syncytiotrophoblast of every sample but no significant differences between groups (**Supplementary Figure 3**). Meanwhile, neuraminidase 1 (NEU1), a member of the human sialidases (neuraminidases) family that controls cellular sialic acid contents in collaboration with sialyltransferases by catalyzing the removal of sialic acid moieties from glycoproteins and glycolipids, was also investigated. Among three sialyltransferases, ST3GAL6 showed the strongest expression, it was prominently higher in the NC group than the RM group (*P <*0.05), but not the SM group (**Figure 5**). Expression of ST3GAL3, ST3GAL4, and FUT4 showed no significant differences among groups (**Figure 5**). NEU1 in the SM group but not the NC group was prominently upregulated than the RM group (*P <*0.05, **Figure 5**). Negative controls are shown in **Supplementary Figure 4**.

### Positive Correlation of ST3GAL6 with sLeX and LeY

**Figure 6:**
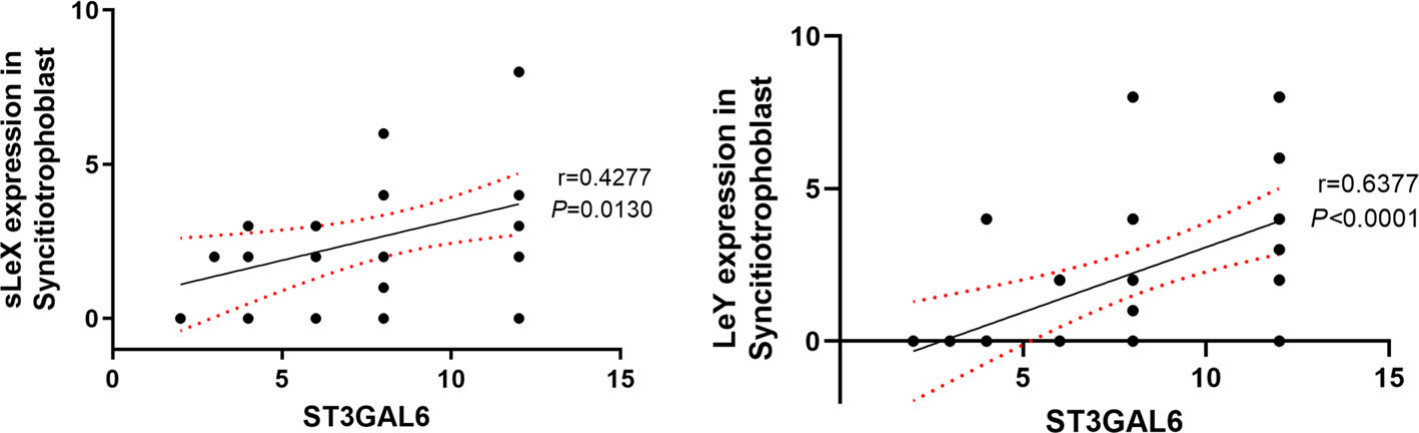
Correlation of sLeX and LeY expression with ST3GAL6 in the syncitiotrophoblast. The IRS scores of sLeX and LeY staining were positively correlated with ST3GAL6 expression in the syncitiotrophoblast. Lines represent the Spearman’s rank correlation (black line) and 95% confidence interval (red dotted line).

Significant correlations were found in the expression of ST3GAL6 with sLeX and LeY (**Table 3** and **Figure 6**). In detail, both sLeX and LeY were positively correlated with ST3GAL6 in the syncytiotrophoblast (*r* = 0.4277, *P* = 0.0130; *r* = 0.6377, *P <*0.0001 respectively). In contrast, there were no prominent correlations of NEU1 with any of the four Lewis antigens (**Table 3**).

**Table 3:** Spearman’s rank correlation coefficients of the expression of ST3GAL6 and sLeA, sLeX, LeX, and LeY.

	ST3GAL6			NEU1		
	*r*	*P* value	95% CI	*r*	*P* value	95% CI
**sLeA**	0.2336	0.1837	−0.1238 to 0.5373	−0.1606	0.3719	−0.4857 to 0.2035
**sLeX**	0.4277	**0.0130**	0.08843 to 0.6780	0.1083	0.5486	−0.2540 to 0.4439
**LeX**	0.2234	0.2190	−0.1464 to 0.5385	−0.07437	0.6858	−0.4213 to 0.2915
**LeY**	0.6377	**<0.0001**	0.3678 to 0.8085	0.05042	0.7841	−0.3133 to 0.4013

R, correlation coefficient; CI, confidence interval. P < 0.05 is in bold and considered to be statistically significant.

### Diminished Villous Vessels in the Miscarriage Group

**Figure 7:**
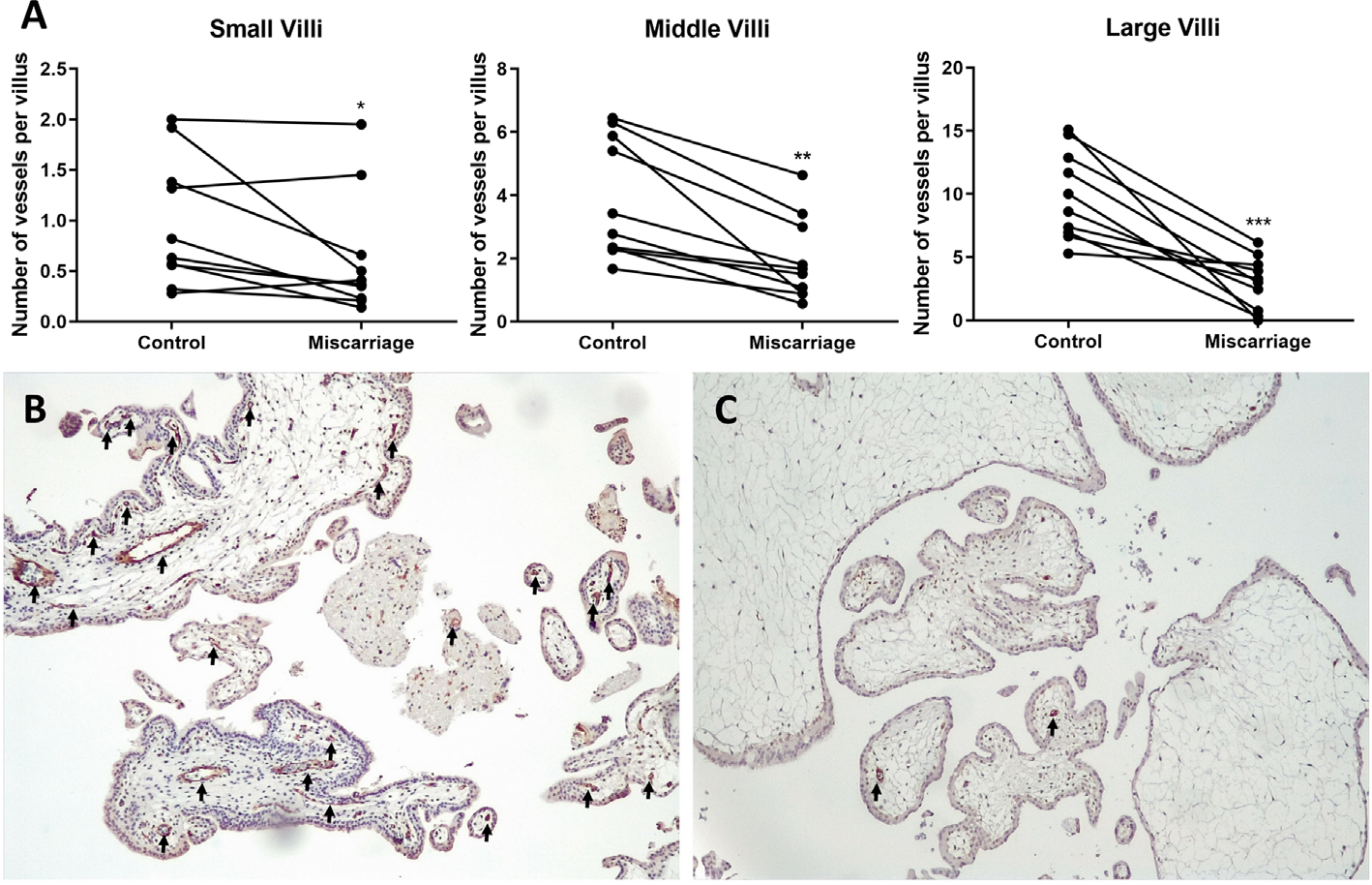
Immunohistochemical staining of CD31 in gestation age-paired placenta tissues (n = 10 each group). **(A)** Paired t-test (for Middle and Large Villi) and Wilcoxon matched-pairs signed rank test (for Small Villi) of vessels’ numbers in different size groups of villi. **(B)** Placenta of Normal Control group (8 + 2 weeks, 100× magnification). **(C)** Placenta of Miscarriage group (8 + 3 weeks, 100× magnification). Black arrows: vessels. *P < 0.05, **P < 0.01, ***P < 0.001.

CD31 staining of gestation ages-paired placenta tissues revealed significantly diminished vessels in the villi of the miscarriage group, which was consistent across all types of villi (**Figure 7**). In the miscarriage group, the mean number of vessels per small villi was 0.62 ± 0.59 (*vs* 0.98 ± 0.63, *P* = 0.0371), per middle villi (1.95 ± 1.32 *vs* 3.88 ± 1.90, *P* = 0.0010), and per large villi (2.95 ± 2.01 *vs* 9.92 ± 3.50, *P* = 0.0003).

### Existence of LeX/Y in the Endothelial Cells of Villous Vessels

**Figure 8:**
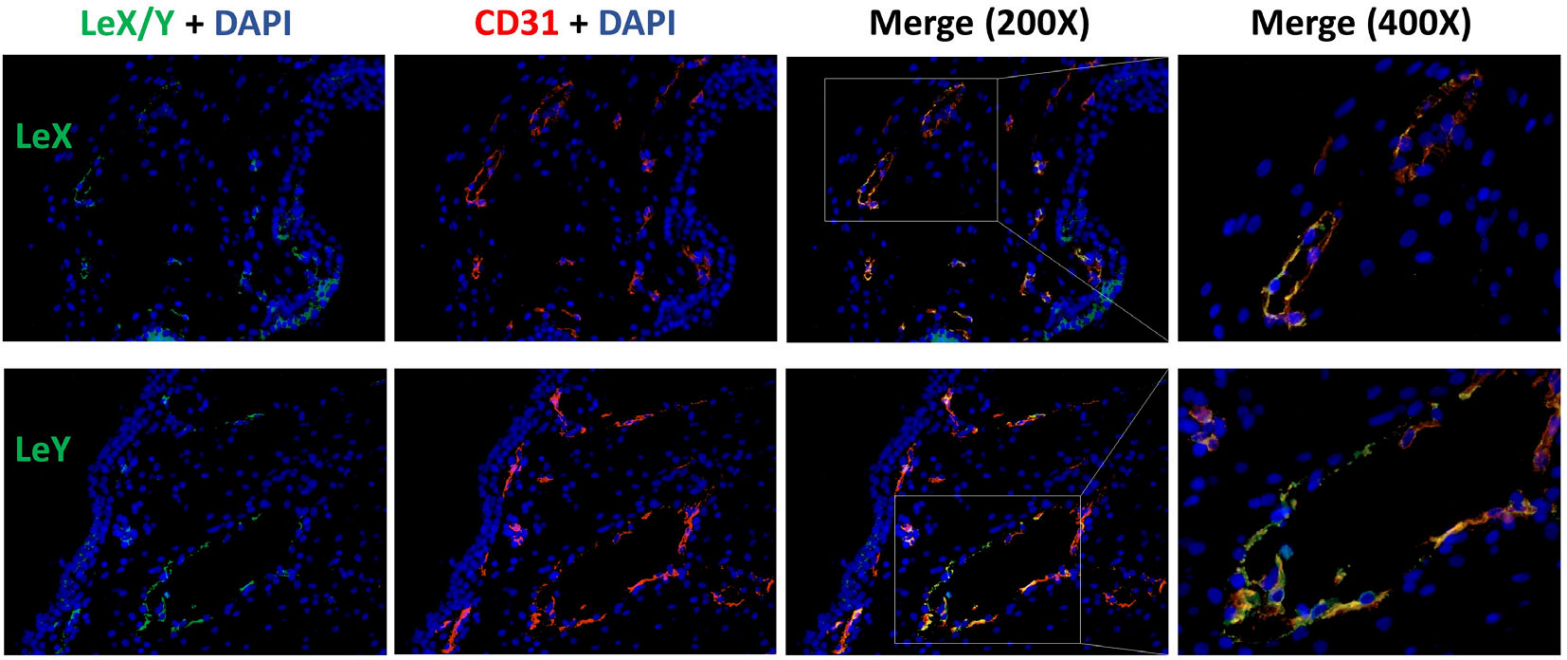
Immunofluorescent double staining of LeX/Y + CD31 in the endothelial cells of villous vessels.

LeX/Y positive cells were stained in green, CD31 positive endothelial cells were red (**Figure 8**). LeX/Y + CD31 double immunofluorescent staining was used to further prove that immunohistochemical staining of LeX/Y inside the villi (**Figure 4**) was exact the endothelial cells of villous vessels. The merged pictures in **Figure 8** confirm the co-localization of LeX/Y and CD31.

## Discussion

Aberrant glycosylation often indicates the tissue inflammation and neoplasia. Particularly, increased sialylation actuates the key processes of tumor progression and metastasis like cell adhesion, invasion, and immune escape (18–22). Meanwhile, there is increasing evidence that altered glycosylation is important in human gamete binding and embryo implantation (8, 11, 23–26). Establishment of balanced maternal immune tolerance towards the semi-allogenic fetus is a crucial step in maintaining a healthy pregnancy. In particular, an inflammatory microenvironment is required for early implantation (27). However, an excessive inflammatory response would lead to RM and other pregnancy complications like pre-eclampsia and premature labor (28). Whether glycosylation alterations interact with inflammatory response and contribute to the pathogenesis of miscarriage is largely unknown. In this study, we report significant downexpression of glycosylation-related signatures and upregulated inflammatory response, which is in line with previous studies (29, 30), in RM through GSEA. Gene enrichment and network analysis performed with downregulated GO_GLYCOSYLATION genes in RM deciphered the significant relationships of aberrant glycosylation with immune system process and biological adhesion, the possible interactions were also visualized (**Figure 2** **)**. Immunohistochemical staining further indicated that Lewis antigens were downregulated in miscarriage groups and key modulators including, ST3GAL6 and NEU1, might be responsible for these alterations.

SLeA, also known as carbohydrate antigen 19-9 (CA19-9), and sLeX are the most common sialylated cell surface glycoconjugates, which mediate the adhesion of leukocytes to endothelial cells and platelets (23). They have been also widely investigated in many types of cancers, and sLeA and sLeX were reported to promote metastasis and malignant transformation (7). SLeA and sLeX were both found to be abundant at the human endometrium during the implantation stage (23). Meanwhile, sLeX was also identified at the level of human zona pellucida, indicating its pivotal role in sperm–egg binding and subsequent adhesion of the embryo to the endometrium (8, 11, 25). Upregulation of sLeX *via* FUT7 transfection promoted the embryo adhesion and implantation were shown in both *in vitro* and *in vivo* model (31, 32). The significantly downregulated sLeA and sLeX in the miscarriage groups revealed by our study may partly explain disturbed trophoblast adhesion and invasion ability during early placentation that leads to subsequent early pregnancy loss.

LeX, also Stage-specific embryonic antigen-1 (SSEA-1), is playing a gradually role in human embryogenesis, especially in cell-cell recognition and adhesion processes which is critical on the surface of embryonic ectodermal cells (33). In the brain, LeX predominantly facilitates the cell-cell interactions involved in neuronal development (34). Moreover, LeX is necessary for neutrophil transepithelial migration (35). On the other hand, overexpression of LeX is usually associated with decreased survival, metastasis, and malignant transformation in many types of cancers (36–40). In accordance with a previous study (41), we also found a weak expression manner of LeX in the syncytiotrophoblast in the NC group, but almost no expression in the miscarriage groups. Given the important role of LeX in the processes of adhesion and metastasis, we speculate that downregulated LeX in the syncytiotrophoblast may contribute to miscarriage for insufficient trophoblast function.

As previously described, LeY has been found in human uterine epithelial tissues and its expression was significantly upregulated during the secretory stages of the menstrual cycle in humans (42). Blocking LeY reduced the adhesion of the human trophoblast cell line (JAR) to the uterine epithelial cell line (RL95-2) in an *in vitro* implantation model (10). Here, we report that both LeY and LeX are expressed in the chorionic villi, and their expression is significantly downregulated in the syncytiotrophoblast of miscarriage groups comparing with the NC group. In addition, LeX and LeY expression was also identified inside the villi in the NC group, but not prominent in the miscarriage group. We further showed that LeX and LeY specifically localized in the endothelial cells of the villous vessel. The villous vessels in the miscarriage group were significantly diminished in all sizes of villi compared with the control group. Candelier et al. and Ziganshina et al. reported similar findings in their work on hydatidiform moles and fetal growth restriction (41, 43). Moreover, the role of LeY in endothelial tube formation and angiogenesis has been previously demonstrated in human rheumatoid arthritis and rat cornea (44, 45). Deficient vascularization has been reported in miscarriages and hydatidiform mole, especially the empty sac miscarriages (46, 47). While Reus et al. showed that there was no prominent difference between empty sac or yolk sac miscarriages and embryonic miscarriages concerning the chorionic villous vascularization (48). In our network analysis, VEGFA-VEGFR2 signaling pathway, which plays a prominent role in the angiogenesis, also interacts with aberrant glycosylation. Thus, the downregulated LeY expression may account for the insufficient trophoblast function as well as the defective vascularization in miscarriages.

Leukocyte infiltration in the decidua is a cardinal feature during first trimester pregnancy, which comprises mainly the natural killer cells, macrophages and, comparatively a few T cells (49). The crosstalk between trophoblast and decidual leukocytes *via* complicated cytokine network allows these two parts to attract each other (49). Alterations in leukocytes recruitment and activation have been extensively related to miscarriage (50). Similarly, our results obtained through network analysis indicated that the aberrant glycosylation might be involved in leukocyte adhesion and migration in RM. Moreover, five key modulators in the synthetic pathways of Lewis antigens were investigated, among which ST3GAL6 and NEU1 showed dysregulated expression. To our knowledge, this is the first description of α-2,3 sialyltransferases, neuraminidase, and fucosyltransferase expression patterns in the chorionic villi of patients with unexplained miscarriages.

ST3GAL3, ST3GAL4, and ST3GAL6 all belong to the α-2,3 sialyltransferases family. While having different substrate specificity, they all catalyze the transfer of sialic acid residues to galactopyranosyl residue (51). *In vitro* study suggested that ST3GAL3 preferentially acts on type I disaccharides (Galβ1,3GlcNAc, the backbone of sLeA), but also mildly catalyze the sialylation of type II disaccharides (Galβ1,4GlcNAc, the backbone of sLeX) (52). Sasaki et al. reported that ST3GAL4 recombinant protein extracted from a melanoma library showed enzymatic activity towards both type I and type II disaccharides in *in vitro* assays whereas preferentially catalyzed type II substrates in *in vivo* condition (53). While most studies found ST3GAL4 acted on type II disaccharides despite *in vitro* or *in vivo* status (54–57). Similarly, ST3GAL6 mainly catalyzes the transfer of sialic acid residues onto type II disaccharides found on glycoproteins and glycolipids (58, 59). We demonstrated a significant correlation of ST3GAL6 expression with sLeX, which is consistent with previously described exclusive ST3GAL6 effect on type II disaccharides. This is especially important, considering that downregulated ST3GAL6 may play a role in the downregulation of sLeX in the villi of recurrent miscarriages. Notably, we also found a strong positive correlation between ST3GAL6 and LeY expression, which cannot be simply explained by the enzymatic specificity of ST3GAL6 since the synthesis of LeY requires the sequential effect of α-1,2 and α-1,3 fucosyltransferases rather than sialyltransferases. Inhibited sialylation potentially allows greater fucosylation due to competition between fucosyltransferase and sialyltransferase, for the same acceptor substrates (57, 60, 61), downregulated ST3GAL6 should have yielded higher expression of LeX and LeY, which was not found in our study. A possible explanation might be the existence of predominantly impaired fucosyltransferases in the miscarriage villi has not been identified yet, on the other hand, the glycosylation network is far more complex than we have known.

FUT4 was reported in many studies to be vital in controlling the synthesis of LeX and LeY (10, 61, 62), but demonstrated no significant difference among the groups included in our analysis. Gadhoum and Sackstein found that NEU1 predominantly increased LeX expression through desialylation sLeX in the process of human myeloid differentiation (63). NEU4, which is another member of the human neuraminidase family, shows a broader substrate specificity compared with NEU1. Previous study indicates that NEU4 desialylates cell surface sLeA/X to LeA/X without affecting the expression of ST3GAL3, ST3GAL4, FUT3, and FUT7 in colon cancer cells (64). Here, we found significantly higher expression of NEU1 in the SM group comparing with the RM group, but no correlations were identified with Lewis antigens, which may due to its limited sialidase capacity on sLeA/sLeX (64).

To conclude, we report significant downregulation of glycosylation-related signatures and adhesion molecules, as well as upregulated inflammatory response in the chorionic villi of RM through GSEA and KEGG pathway analysis. Accordingly, downregulation of sLeA, sLeX, LeX, and LeY were further identified. Key modulators, such as ST3GAL6 and NEU1 may be involved and account for these alterations. Interestingly, significantly diminished villous vessels were also identified in the miscarriage group. Taken together, our data might indicate that altered expression of Lewis antigens may actuate miscarriages by affecting 1) trophoblast function 2) its interaction with decidual leukocytes and 3) the vascularization of the villi. A better understanding of the vital role that Lewis antigens are playing in pathology of miscarriages will yield the development of novel therapeutic approaches, which still requires more future work.

## Data Availability Statement

The original contributions presented in the study are included in the article/**Supplementary Material**. Further inquiries can be directed to the corresponding author.

## Ethics Statement

The studies involving human participants were reviewed and approved by the Ethics Committee of the LMU Munich. The patients/participants provided their written informed consent to participate in this study.

## Author Contributions

UJ and VS conceived of the study and participated in its design and coordination. ZM performed the experiments and statistical analysis and wrote the manuscript. HY, LP, and CK performed technical assistance in immunohistochemistry assays. HY, LP, CK, AC-R, SM, UJ, and VS revised the manuscript for important intellectual content. All authors contributed to the article and approved the submitted version.

## Conflict of Interest

The authors declare that the research was conducted in the absence of any commercial or financial relationships that could be construed as a potential conflict of interest.

The handling Editor declared a past co-authorship with one of the authors (UJ).
