# Supplementary figures and images for: Expression of the Carbohydrate Lewis Antigen, Sialyl Lewis A, Sialyl Lewis X, Lewis X, and Lewis Y in the Placental Villi of Patients With Unexplained Miscarriages
Source: Front Immunol. 2021 May 31;12:679424. doi: 10.3389/fimmu.2021.679424 (PMC8202085; doi:10.3389/fimmu.2021.679424)

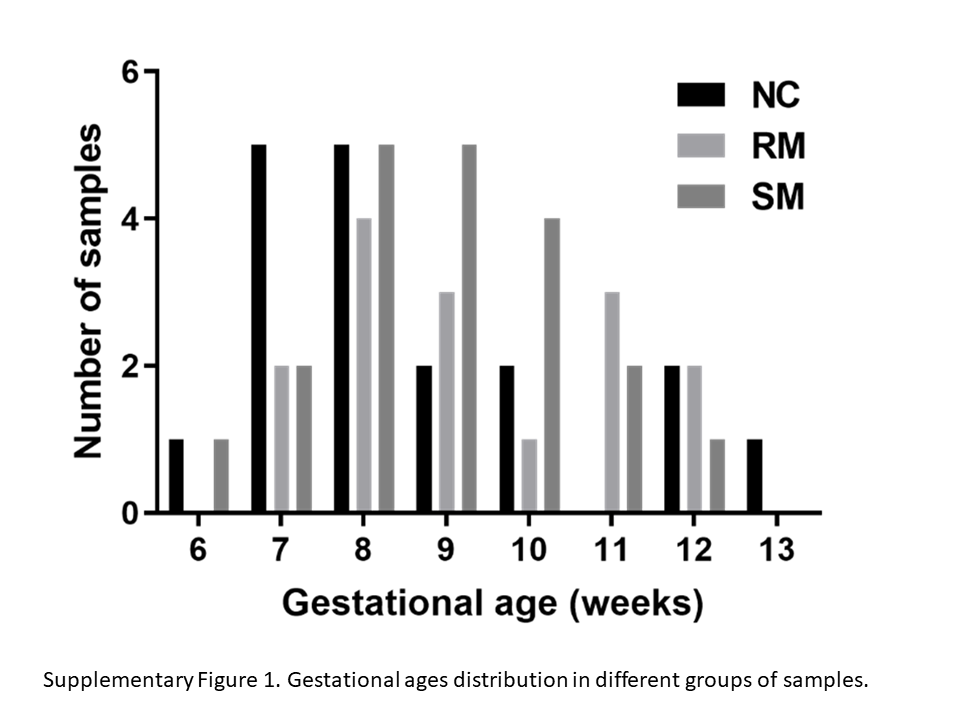

Supplement: Supplementary file 1 [file Image_1.tif]

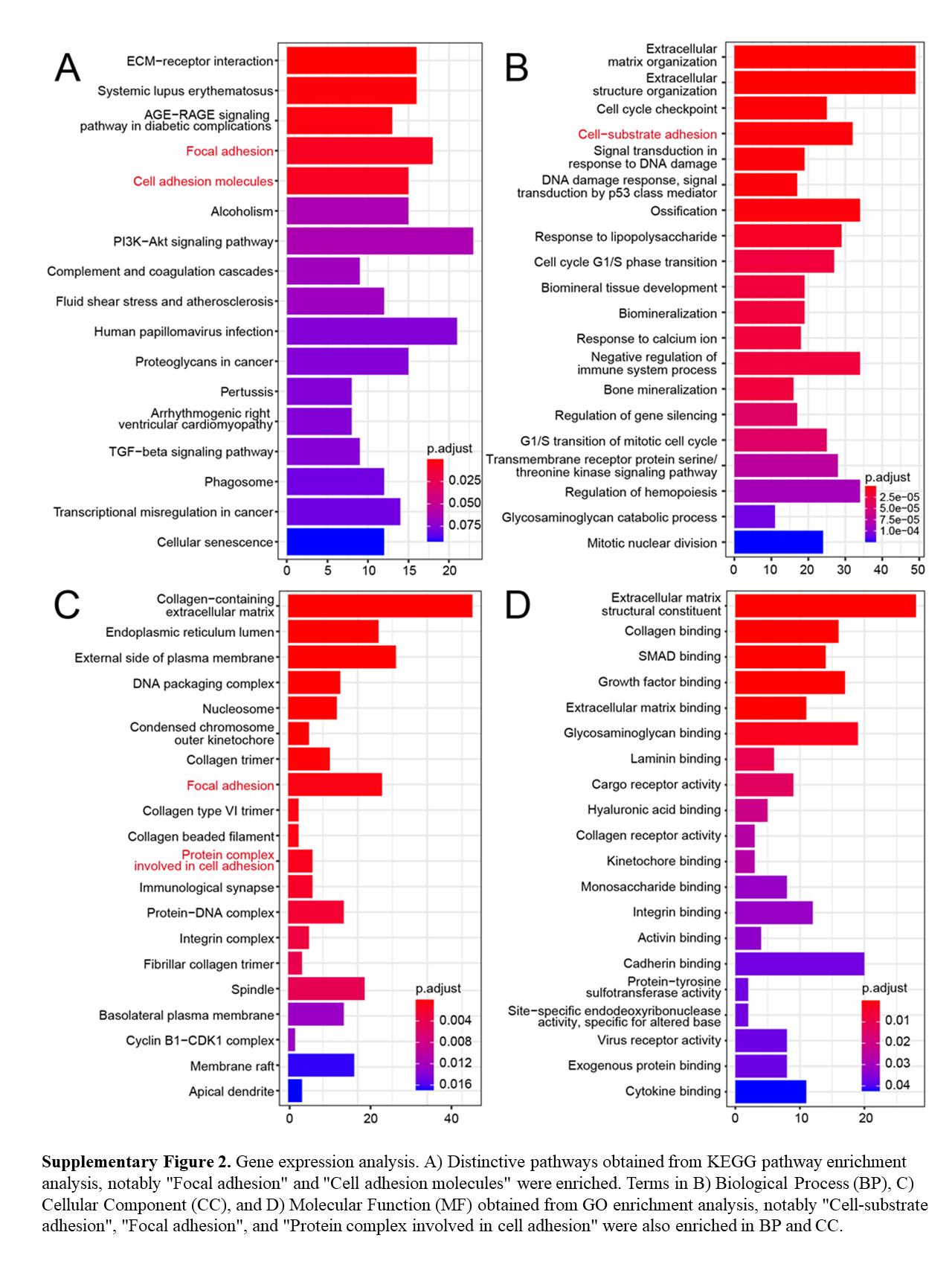

Supplement: Supplementary file 2 [file Image_2.tif]

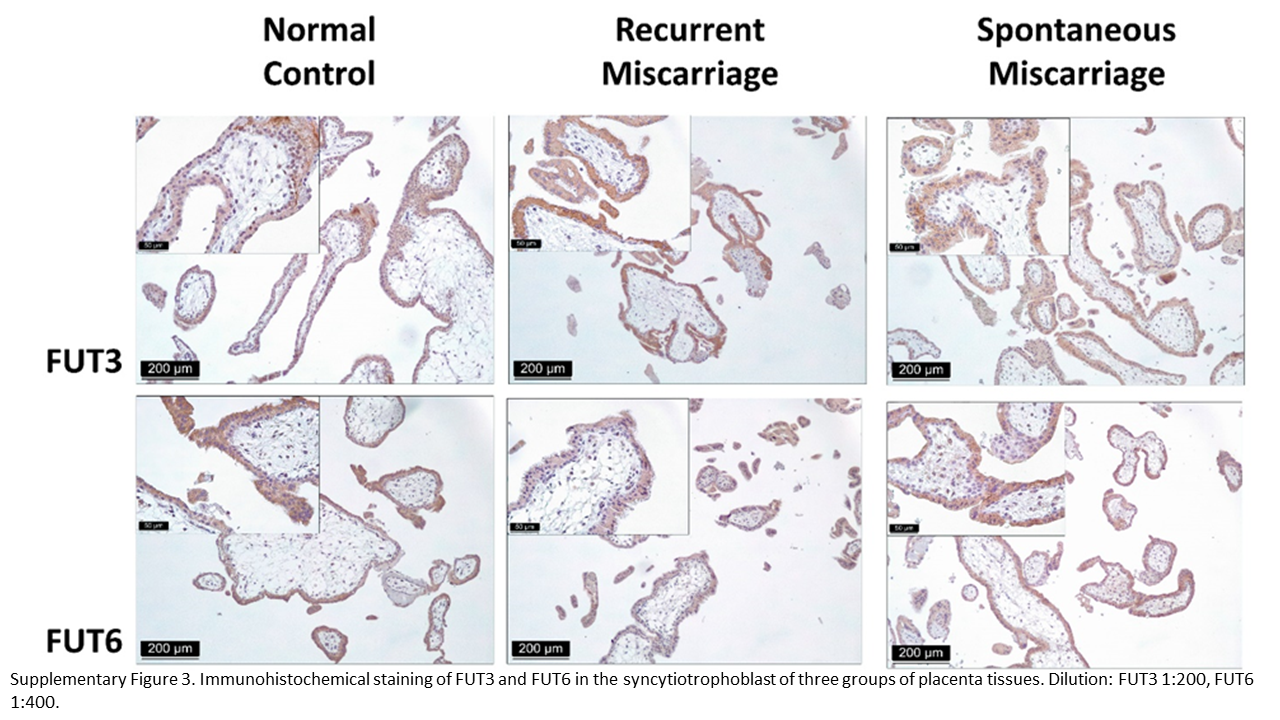

Supplement: Supplementary file 3 [file Image_3.tif]

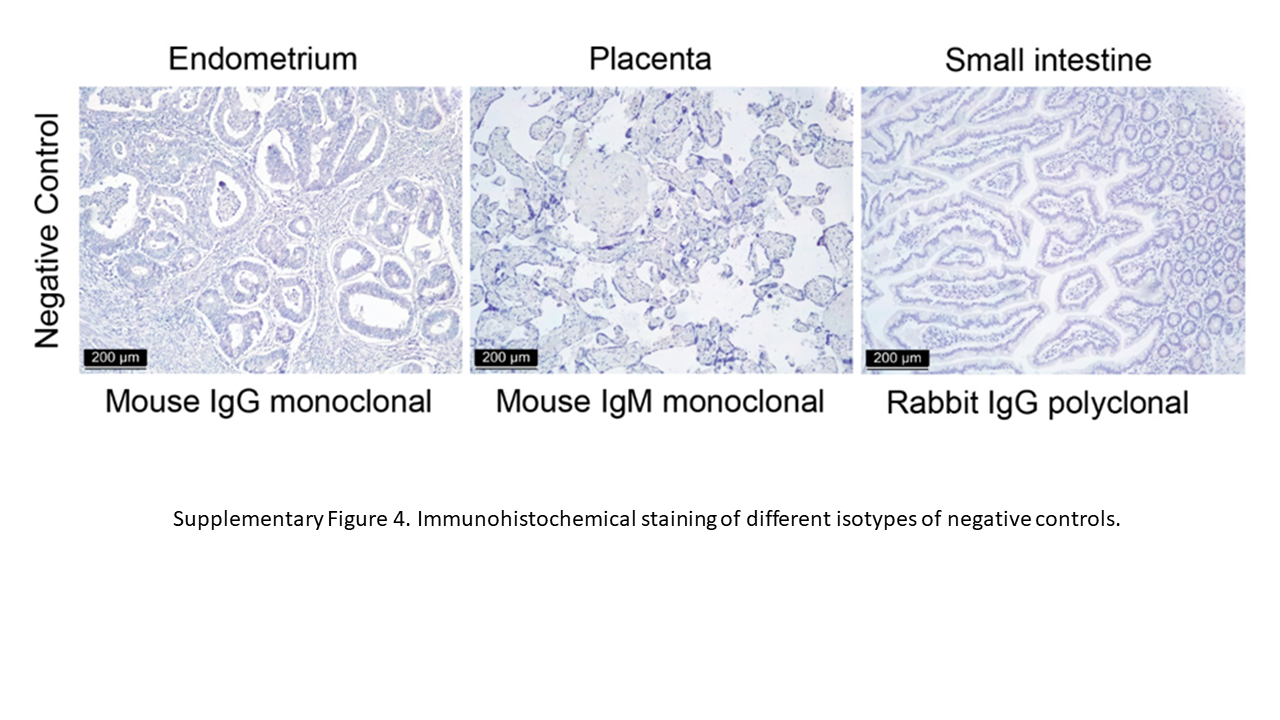

Supplement: Supplementary file 4 [file Image_4.tif]
